# Hydrochloric Acid in Small-Pox

**Published:** 1860-04

**Authors:** Wm. McDonald


*Treatment of Small-Pox by Hydrochloric Acid.*—I beg to call the attention of the profession to the great value of hydrochloric acid in both the external and internal treatment of small-pox. It allays the prickling pain so distressing in some cases, reduces the tumefaction, the vesicle maturates earlier, and desquamation takes place sooner, leaving the skin smoother and purer than by any other plan which I have tried. Internally, one drachm of the commercial acid to twelve ounces of water; dose, a tea-spoonful in a glass of water; to be sipped often. Externally, I use it to the face, hands, and feet—the parts which suffer most from irritation; for the face, half a drachm to, say, ten ounces of water: apply with a hair pencil twice or thrice daily, using occasionally the mercurial liniment or cold cream. If the cuticle on the extremities be hard and horny, it may be used stronger. I shall not here enter into an account of its specific action, as I am preparing a paper on its use in various diseases, which, with your kind permission, I shall shortly publish in your valuable journal. The present epidemic of small-pox affords ample scope for its trial, and I am confident that it will be as serviceable in the hands of my professional brethren as in mine.—*Dr. Wm. McDonald, in Lancet.*

